# Supplementary material for: Intracellular pH dynamics respond to extracellular matrix stiffening and mediate vasculogenic mimicry through β-catenin
Source: Cell Death Dis. 2025 Oct 13;16(1):720. doi: 10.1038/s41419-025-08014-z (PMC12518539; doi:10.1038/s41419-025-08014-z)
Supplement: Supplementary file 1 — Supplemental Figures 1-9 [file 41419_2025_8014_MOESM1_ESM.pdf]

Supplementary Materials for

Intracellular pH dynamics respond to extracellular matrix stiffening and mediate vasculogenic mimicry through β-catenin

Leah M. Lund[1,2], Angelina N. Marchi[1,2,5], Laura Alderfer[3,6], Eva Hall[3], Jacob Hammer[1,2], Riley Moremen[1,2], Ijeoma Asilebo[1,2], Keelan J. Trull[1,2,7], Donny Hanjaya-Putra[2,3,4] and Katharine A. White[1,2*]

*Corresponding author. Email: kwhite6@nd.edu

This PDF file includes:

Supplementary Figures S1 to S9

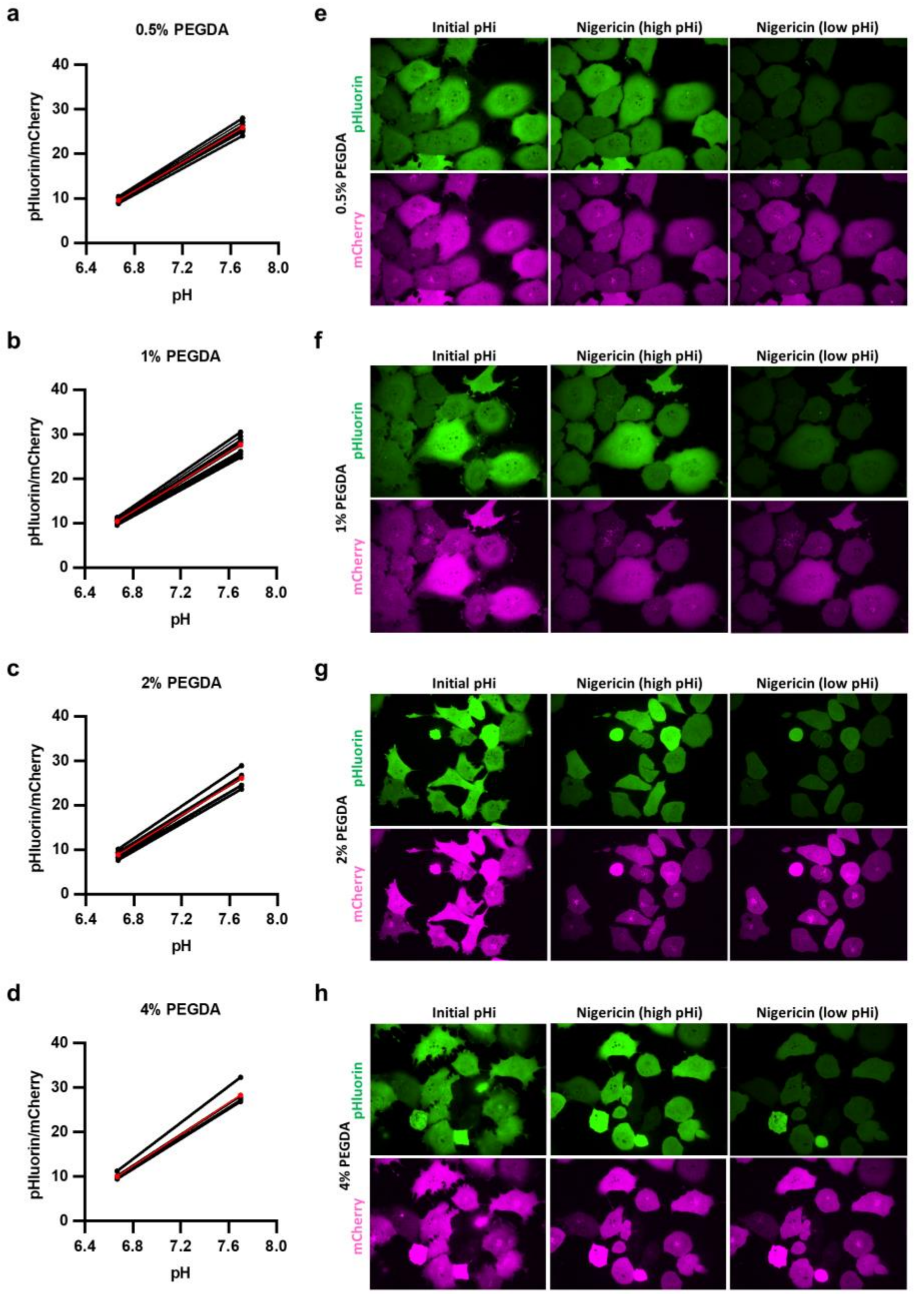

Figure S1: Nigericin standardization of mCherry-pHluorin biosensor in H1299 cells. Nigericin standard linear regressions across a single replicate for each single cell (black) and mean (red) for H1299-mCherry-pHluorin plated on **a)** 0.5% PEGDA (n=10), **b)** 1% PEGDA (n=10), **c)** 2% PEGDA (n=13), **d)** 4% PEGDA (n=7) HA gels. Single channel images of pHluorin (green) and mCherry (magenta) fluorescence at initial pHi imaging, high nigericin, and low nigericin standardizations for **e)** 0.5% PEGDA, **f)** 1% PEGDA, **g)** 2% PEGDA, **h)** 4% PEGDA. LUTs are identical across each cell line. Note that in all cases, single-cell nigericin standard curves were used to back-calculate single-cell pHi.

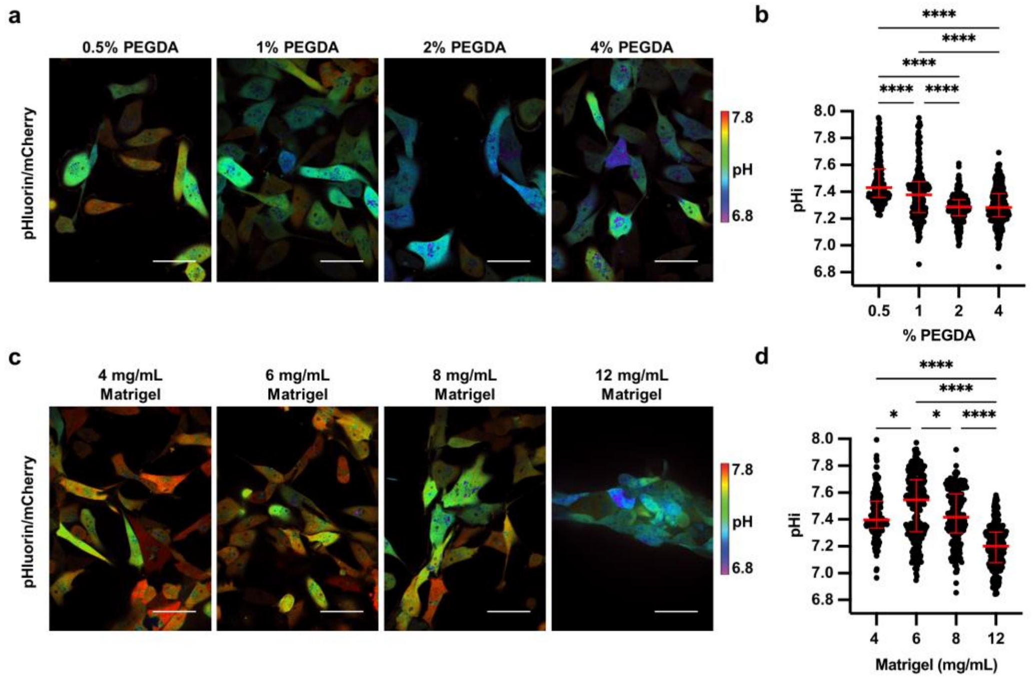

Figure S2: Stiffening extracellular matrix lowers pHi in metastatic human breast carcinoma (MDA-MB-231). a) Representative images of MDA-MB-231 cells stably expressing mCherry-pHluorin pH biosensor plated on varying HA gel stiffnesses. Images show ratiometric display of pHluorin/mCherry fluorescence. Scale bars: 50 µm. **b)** Quantification of single-cell pHi data collected as shown in (a) (n=3 biological replicates, n=194 0.5% PEGDA, n=228 1% PEGDA, n=265 2% PEGDA, n=334 4% PEGDA. Red lines show medians ± IQR). **c)** Representative images of MDA-MB-231 cells stably expressing mCherry-pHluorin pH biosensor plated on varying Matrigel stiffnesses. Images show ratiometric display of pHluorin/mCherry fluorescence. Scale bars: 50 µm. **d)** Quantification of single-cell pHi data collected as shown in (c) (n=3 biological replicates, n=210 4mg/mL, n=291 6mg/mL, n=222 8mg/mL, n=292 12mg/mL. Red lines show medians ± IQR). For (c) and (d), significance was determined by a Kruskal-Wallis test (*P<0.05; **P<0.01; ***P<0. 001; ****P<0.0001).

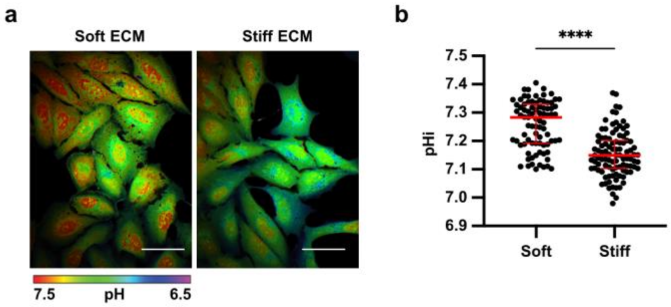

Figure S3: Stiffening extracellular matrix increases pHi in metastatic bone cells (U-2 OS). a) Representative images of U-2 OS cells loaded with SNARF pH dye plated on soft (0.5% PEGDA) and stiff (4% PEGDA) HA gels. Images show ratiometric display of SNARF/TxRed fluorescence ratios. Scale bars: 50 µm. **b)** Quantification of single-cell pHi data collected as shown in (a) (n=4 biological replicates; n=299 soft, n=305 stiff. Red lines show medians ± IQR). For (b), significance was determined by a Kruskal-Wallis test (*P<0.05; **P<0.01; ***P<0. 001; ****P<0.0001).

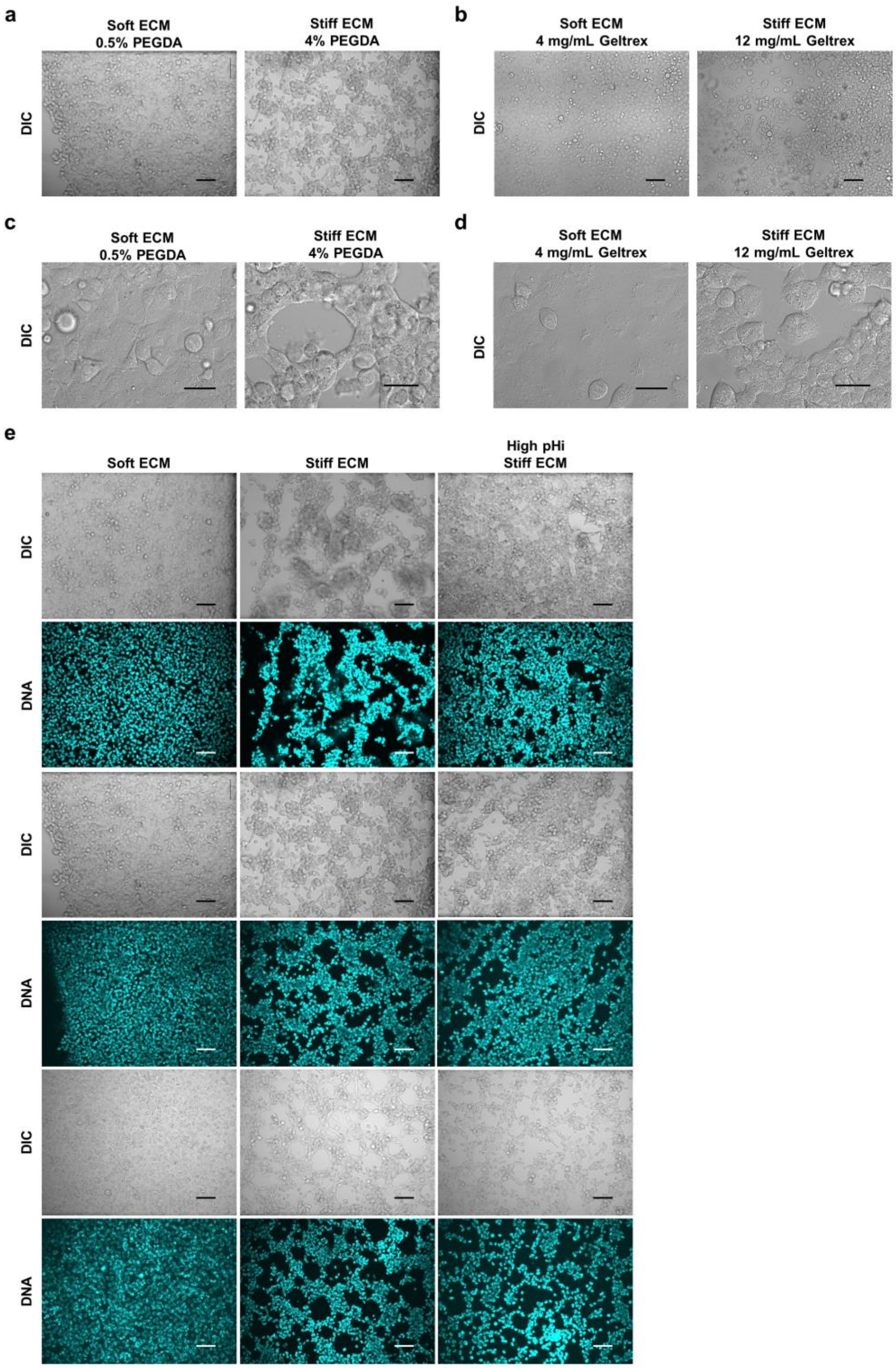

Figure S4: Representative images of Vasculogenic mimicry phenotype on soft and stiff Geltrex and HA gels. a) Differential interference contrast (DIC) images of H1299 cells plated on soft (0.5% PEGDA) and stiff (4% PEGDA) HA gels. Scale bars: 100 μm. **b)** Differential interference contrast (DIC) images of H1299 cells plated on soft

(4mg/mL) and stiff (12 mg/mL) Geltrex gels. Scale bars: 100 μm. **c)** Differential interference contrast (DIC) images of H1299 cells plated on soft (0.5% PEGDA) and stiff (4% PEGDA) HA gels. Scale bars: 50 μm. **b)** Differential interference contrast (DIC) images of H1299 cells plated on soft (4mg/mL) and stiff (12 mg/mL) Geltrex gels. Scale bars: 50 μm. **e)** Differential interference contrast (DIC) and Hoechst stain (DNA, cyan) images of H1299 cells plated on soft (0.5% PEGDA), stiff (4% PEGDA), and stiff (4% PEGDA) with high pHi HA gels. Scale bars: 100 μm.

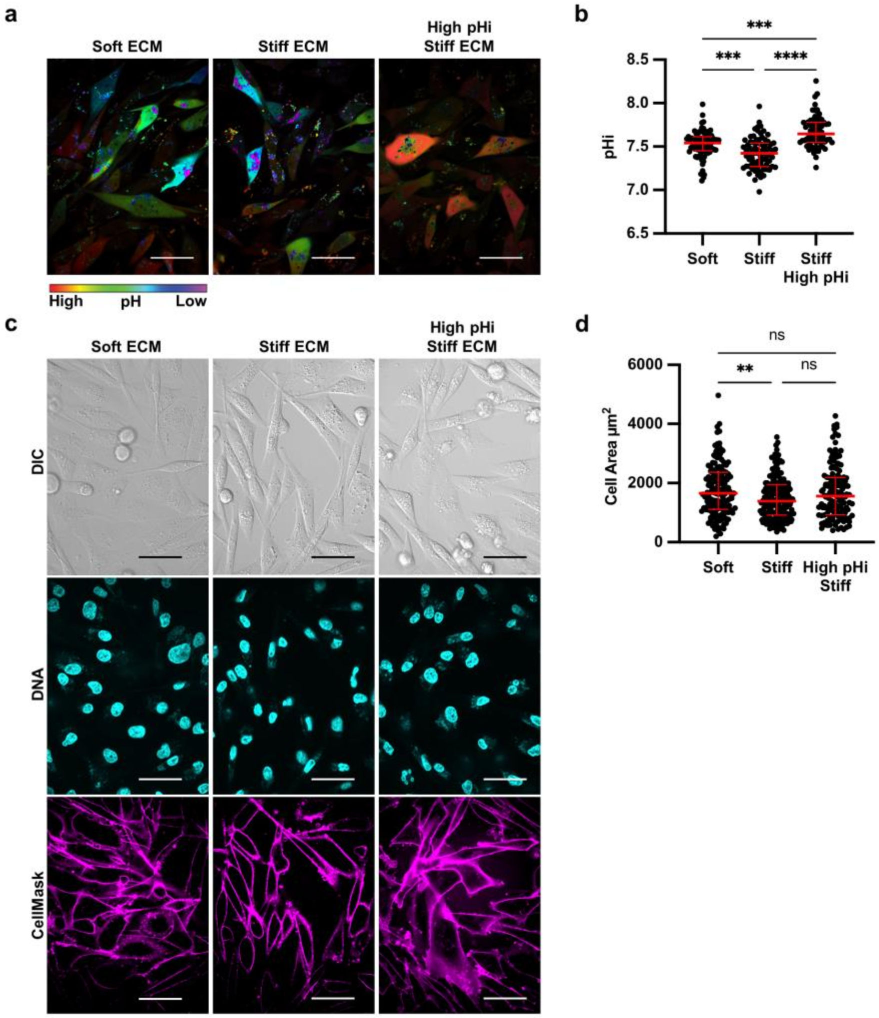

Figure S5: Stiffness-dependent vasculogenic mimicry is reduced when pHi is increased on stiff ECM in metastatic breast cells (MDA-MB-231). a) Representative images of MDA-MB-231 cells stably expressing mCherry-pHluorin pH biosensor plated on soft (0.5% PEGDA) and stiff (4% PEGDA) HA gels and stiff (4% PEGDA) with raised pHi (culture media supplementation with 50mM sodium bicarbonate). Images show ratiometric display of pHluorin/mCherry fluorescence ratios. Scale bars: 50 µm. **b)** Quantification of single-cell pHi data collected as shown in (a) (n=2 biological replicates; n=79 soft, n=81 stiff, n=69 stiff high pHi. Red lines show medians ± IQR). **b)** Representative images of MDA-MB-231 cells plated on soft (0.5% PEGDA) and stiff (4% PEGDA) HA gels and stiff (4% PEGDA) with raised pHi. Images show differential interference contrast (DIC), Hoechst 33342 (DNA, cyan) and CellMask Deep Red membrane stain (Cy5, magenta). Scale bars: 50 µm. **c)** Quantification of single-cell area collected as shown in (b) (n=2 biological replicates, n=151 soft, n=175 stiff, n=132 stiff high pHi. Red lines show medians ± IQR). For (b) and (d), significance was determined by a Kruskal-Wallis test (**P*<0.05; ***P*<0.01; ****P*<0. 001; *****P*<0.0001).

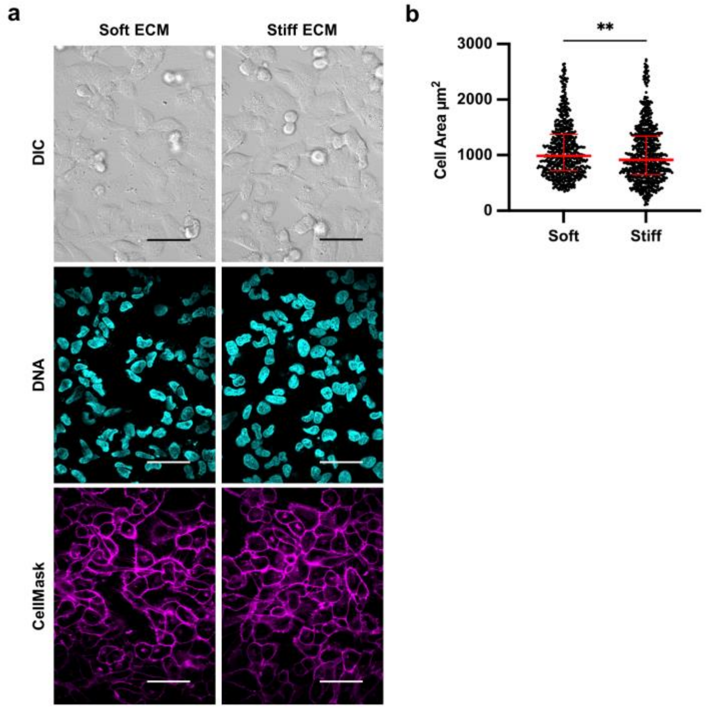

Figure S6: Stiffness-dependent vasculogenic mimicry is reduced when pHi is increased on stiff ECM in metastatic bone cells (U-2 OS). a) Representative images of U-2 OS cells plated on soft (0.5% PEGDA) and stiff (4% PEGDA) HA gels. Images show differential interference contrast (DIC), Hoechst 33342 (DNA, cyan) and CellMask Deep Red membrane stain (Cy5, magenta). Scale bars: 50 µm. **c)** Quantification of single-cell area collected as shown in (a) (n=2 biological replicates, n=855 soft, n=836 stiff. Red lines show medians ± IQR). For (b), significance was determined by a Kruskal-Wallis test (**P*<0.05; ***P*<0.01; ****P*<0. 001; *****P*<0.0001).

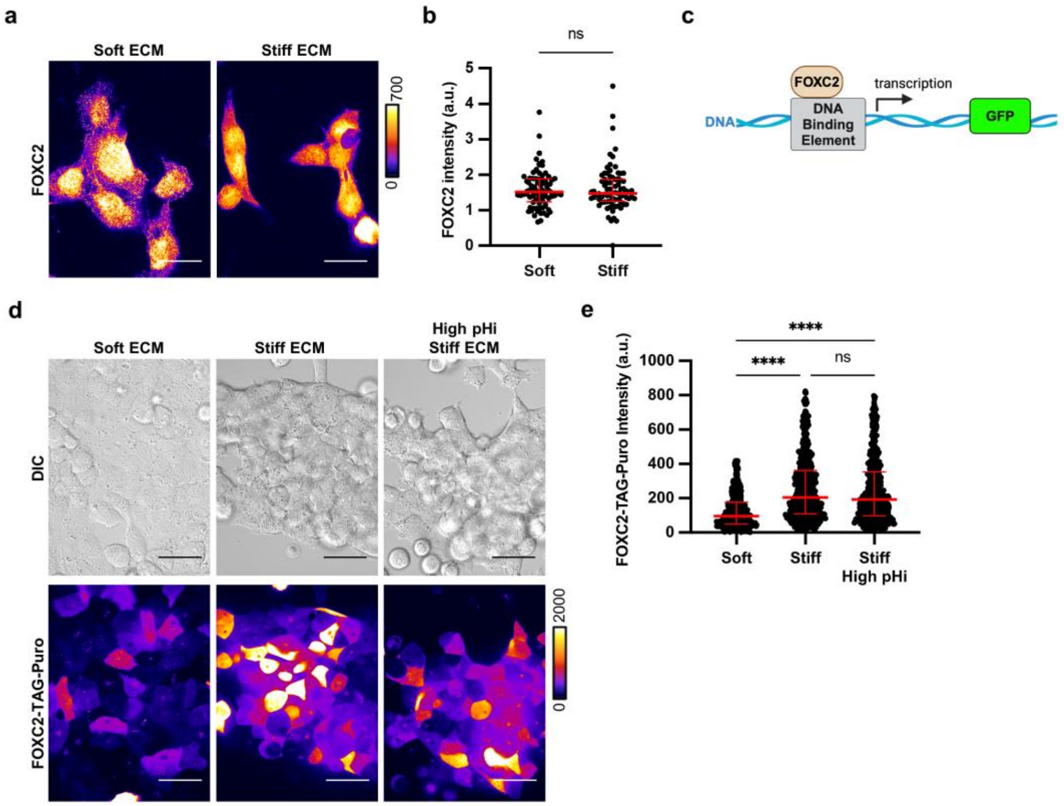

Figure S7: FOXC2 has stiffness-dependent activity that is insensitive to pHi. a). Representative images of H1299 cells plated on soft (4 mg/mL) and stiff (12 mg/mL) Geltrex fixed and stained for FOXC2. FOXC2 is pseudocolored according to scale. Scale bars: 50 µm. **b)** Quantification of FOXC2 intensity per cell collected as shown in (a) (n=3 biological replicates, n=80 soft and n=77 stiff. Red lines show medians ± IQR). **c)** Schematic of FOXC2-TAG-Puro reporter of FOXC2 transcriptional activity. **d)** Representative images of H1299 cells plated on soft (0.5% PEGDA), stiff (4% PEGDA) and stiff (4% PEGDA) with raised pHi HA gels. Images show Brightfield display (DIC) and FOXC2-TAG-Puro. FOXC2-TAG-Puro is pseudocolored according to scale. Scale bars: 50 µm. **e)** Quantification of FOXC2-TAG-Puro intensity per cell collected as shown in (d) (n=3 biological replicates, n=416 soft, n=478 stiff, n=461 stiff high pHi. Red lines show medians ± IQR). For (b) and (e), significance was determined by a Kruskal-Wallis test (**P*<0.05; ***P*<0.01; ****P*<0. 001; *****P*<0.0001).

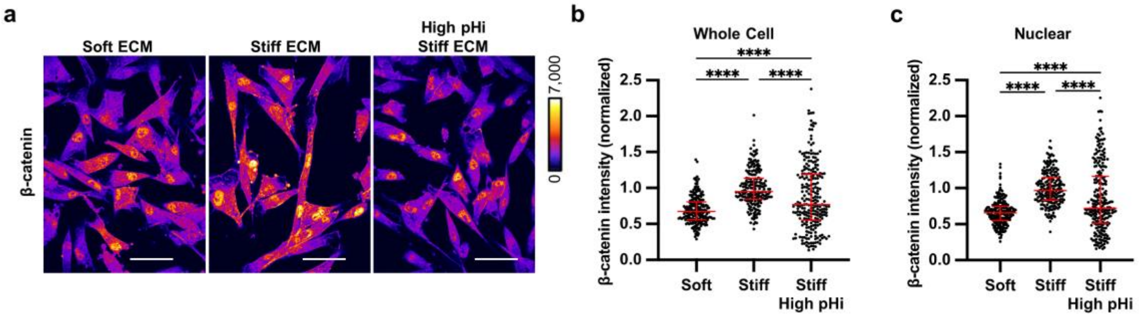

Figure S8: Increased pHi reduced β-catenin abundance and nuclear localization in stiff matrix conditions. a) Representative images of MDA-MB-231 cells plated on soft (0.5% PEGDA), stiff (4% PEGDA) and stiff with raised pHi (4% PEGDA) HA gels fixed and stained for β-catenin. β-catenin is pseudocolored according to scale. Scale bars: 50 μm. **b)** Quantification of whole cell β-catenin intensity collected as shown in (a). (n=3 biological replicates, n=208 soft, n=205 stiff, n=245 stiff high pHi. Red lines show medians ± IQR). **c)** Quantification of nuclear β-catenin intensity collected as described in (a). (n=3 biological replicates, n=208 soft, n=204 stiff, n=244stiff high. Red lines show medians ± IQR). For (b) and (c), significance was determined by a Kruskal-Wallis test (**P*<0.05; ***P*<0.01; ****P*<0. 001; *****P*<0.0001).

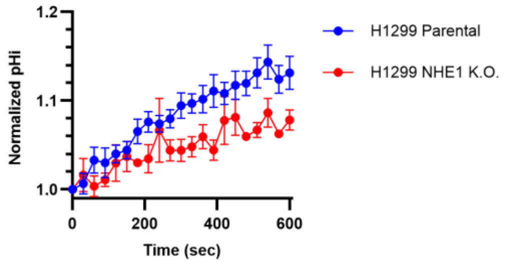

Figure S9: NHE1 activity is decreased in H1299 cells with CRISPR knockout of NHE1. Normalized pHi recovery measurements after acid load of parental H1299 cells (see methods) and H1299 cells where NHE1 has been removed via CRISPR (H1299-NHE1 K.O., see methods). (n=4 biological replicates; n=21 H1299 parental, n=21 H1299 NHE1 K.O.); (means ± SEM).
